# LAG3 constrains anti-parasitic response by effector CD4^+^ T-cell in early *Echinococcus multilocularis*-infected mice

**DOI:** 10.1186/s13071-026-07246-y

**Published:** 2026-02-12

**Authors:** Abidan Ainiwaer, Dewei Li, Wenge Liu, Bingqing Deng, Yinshi Li, Wenying Xiao, Sheng Sun, Yi Gao, Na Tang, Conghui Ge, Jing Li, Hui Wang, Chuanshan Zhang

**Affiliations:** 1 Basic Medical College, Xinjiang Medical University, Urumqi, Xinjiang China; 2 State Key Laboratory of Pathogenesis, Prevention and Treatment of High Incidence Diseases in Central Asia, Clinical Medicine Institute, The First Affiliated Hospital of Xinjiang Medical University, Urumqi, Xinjiang China; 3 Center of Respiratory and Critical Care Medicine, People’s Hospital of Xinjiang Uygur Autonomous Region, Urumqi, Xinjiang China

**Keywords:** Alveolar echinococcosis, LAG3, CD4^+^ T cell subsets, Early infection

## Abstract

**Background:**

Immune checkpoint molecules such as lymphocyte activation gene-3 (LAG3) play a critical role in modulating host–pathogen interactions during chronic parasitic infections; however, their functions in early infection remain poorly defined. Using a murine model infected with *Echinococcus multilocularis* (*E. multilocularis*), a lethal helminth causing alveolar echinococcosis (AE), we elucidate the stage-specific regulatory functions of LAG3 in CD4^+^ T cell immunity during early infection.

**Methods:**

*Echinococcus multilocularis*-infected mice were employed as the experimental model. Flow cytometry was used to analyze LAG3 expression on CD4^+^ T cell subsets and the production of intracellular cytokines. The in vivo functional role of CD4^+^ T cells was further investigated using LAG3-knockout mice and adoptive T cell transfer models. Infected wild-type mice received LAG3 blocking antibody treatment, with parasite burden assessed by measuring metacestode weight. Hepatic pathology was evaluated using hematoxylin and eosin and Masson’s trichrome staining.

**Results:**

We found that LAG3 was predominantly expressed on hepatic effector CD4^+^ T (CD4^+^ Teff) cells, particularly the CD69^+^ subset in the early stages of *E. multilocularis* infection. Functional analysis showed that LAG3-expressing CD4^+^CD44^+^ T cells secreted elevated levels of interleukin-4 (IL-4) and IL-10 at 2 and 4 weeks post-infection. LAG3 deficiency further enhanced the production of interferon-y (IFN-γ), IL-4, and IL-10 by splenic CD4^+^ T cells in the initial infection phase, which may contribute to the slight suppression of *E. multilocularis* growth and development observed in the livers of LAG3-knockout mice. In an adoptive transfer model of early *E. multilocularis* infection, LAG3^−/−^CD4^+^ T cells exhibited a greater propensity to differentiate into CD4^+^ effector T (Teff) cells and produced higher levels of IFN-γ and IL-10 in both the livers and spleens of recipient mice. Finally, early administration of anti-LAG3 monoclonal antibody (mAb) reduced metacestode burden—though the change was not statistically significant—and concurrently exacerbated hepatic inflammation and fibrosis.

**Conclusions:**

Our study reveals a previously unrecognized role for LAG3 as an immune checkpoint during early *E. multilocularis* infection, highlighting its function in limiting anti-parasitic CD4^+^ T cell responses. Thus, while timed LAG3 blockade may represent a potential therapeutic strategy for AE, our data underscore the critical importance of stage-specific intervention to balance parasitic clearance with the control of fibrosis and tissue damage.

**Graphical Abstract:**

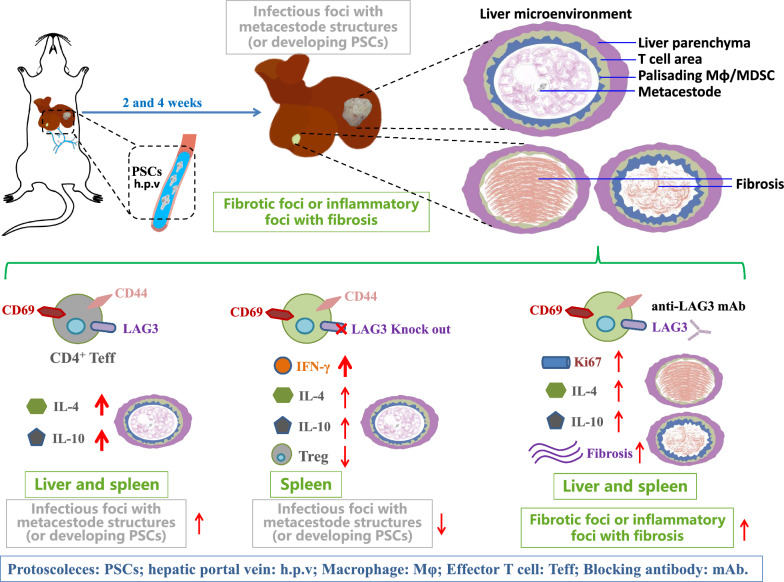

**Supplementary Information:**

The online version contains supplementary material available at 10.1186/s13071-026-07246-y.

## Background

Alveolar echinococcosis (AE) is a highly lethal zoonotic parasitic disease caused by the larval stage (metacestode) of *Echinococcus multilocularis* (*E. multilocularis*), which is predominantly endemic in regions above 40°N latitude [1, 2]. Epidemiological studies confirm significantly higher infection risk in immunocompromised populations compared to healthy individuals [3]. During host–parasite interactions, CD4^+^ T cells have been established as the central hub of immune regulation; clinical and experimental evidence demonstrates that the depletion or functional exhaustion of CD4^+^ T cells markedly accelerates metacestode proliferation [4, 5], whereas targeting T regulatory (Treg) cells or immune checkpoint molecules (e.g., programmed death 1 [PD-1], T cell immunoreceptor with immunoglobulin [Ig] and immunoreceptor tyrosine-based inhibitory motif [ITIM] domain [TIGIT]) on CD4^+^ T cells significantly suppresses lesion progression [5–7]. Notably, the functional contribution of CD4^+^ T cells exhibits distinct stage-dependent dynamics during infection—a critical determinant of parasite immune evasion strategies [4].

CD4^+^ T cell subsets orchestrate infection outcomes through distinct cytokine networks. Early in infection, the T helper type 1 (Th1)-dominant protective immunity: interferon-γ (IFN-γ)-driven Th1 responses activate macrophage-mediated metacestode killing. In rodent models, exogenous administration of Th1 inducers like interleukin-12 (IL-12) significantly reduces parasite colonization [8, 9]. During chronic infection, the T helper type 2 (Th2) and Treg-coordinated tolerance microenvironment: IL-4/IL-13-mediated Th2 responses induce alternatively activated macrophage (M2 macrophage) polarization and synergize with Treg cells to establish an immunosuppressive network, collectively facilitating metacestode survival and proliferation [10]. Notably, during the critical early window of infection (0–4 weeks), hosts exhibit characteristic immune deviation: Th2 markers (e.g., IL-4) surge dramatically within 72 h post-infection, while Th1 effector functions (e.g., IFN-γ) are suppressed [4, 10, 11]. This early immune imbalance is thought to enable the metacestodes to evade host immunity and establish infection.

Lymphocyte activation gene-3 (LAG3, CD223), an emerging immune checkpoint molecule [12], exhibits three pivotal characteristics in *E. multilocularis* infection: (i) stage-dependent expression, with longitudinal increases on LAG3 on CD4^+^ T cells over time [4]; (ii) clinical relevance, as CD4^+^ LAG3^+^ T cell infiltration positively correlates with metacestode lesion volume [4]; and (iii) therapeutic potential in late-stage intervention, wherein LAG3 deficiency enhances Th1 responses and reduces lesion burden [6]. Despite these insights, the role of LAG3 during the early colonization phase—a decisive period for parasite establishment—remains poorly understood.

To address this, we investigated LAG3 function during two pivotal early phases: (i) the acute inflammatory phase (2 weeks), marking initial immune recognition and parasite invasion; and (ii) the colonization establishment phase (4 weeks), during which metacestodes initiate proliferation and tissue localization. We found that LAG3 is predominantly expressed on CD4^+^ Teff and CD69^+^CD4^+^ Teff cells in early infection. In addition, LAG3^+^CD4^+^ Teff cells secrete elevated levels of IL-4 and IL-10, promoting an immunosuppressive environment that facilitates the initial protoscoleces (PSCs) invasion in the liver post-infection. Moreover, LAG3 deficiency enhances antiparasitic immunity, not only by promoting Th1 response in the spleen, but also by strengthening Th2 reactivity and thus contributing to early clearance of *E. multilocularis* infection at 2 weeks post-infection. We also demonstrated that LAG3 blockade significantly increases the area of inflammatory foci with fibrosis and mildly attenuated metacestode growth at 4 weeks. Together with previous findings, this study elucidates a mechanism by which LAG3 modulates CD4^+^ T cell immunity across both early and late stages of *E. multilocularis* infection, highlighting its potential as a stage-specific therapeutic target.

## Methods

### Mice

Age-matched female C57BL/6 wild-type (WT) mice were purchased from Beijing Vital River Experimental Animal Technology Co., Ltd. (Beijing, China). C57BL/6 LAG3-knockout (KO) mice were obtained from Shanghai Model Organisms Center, Inc. (Shanghai, China). C57BL/6 CD4 KO mice were provided by Dr. ZheXiong Lian (Guangdong Academy of Medical Sciences, Guangzhou, China). The mice were raised in a pathogen-free environment of the Animal Experimental Center of Xinjiang Medical University. All the procedures and experiments were approved by the Ethics Committee of the First Affiliated Hospital of Xinjiang Medical University (no. 20170809-01).

### *Echinococcus multilocularis* protoscoleces (PSCs) and infection

PSCs were isolated from the abdominal cavity of infected gerbils in a sterile environment. As previously mentioned, live PSCs in saline (3000 PSCs per mice) were inoculated through the hepatic portal vein of mice, whereas control mice were injected with isotonic saline of equal volume [4].

### Pathological staining

The collected mouse liver tissues were fixed in 4% paraformaldehyde for 48 h. Following fixation, the tissues underwent dehydration and paraffin embedding, and were sectioned at 4-μm thickness. Prior to staining, the sections were dewaxed in xylene for 30 min in two separate sessions, and then rehydrated through a graded ethanol series (100%, 95%, 85%, 80%, 75%) and deionized water for 5 min at each step.

For hematoxylin–eosin (HE) staining, lesions were classified as “fibrotic foci,” “inflammatory foci with fibrosis,” or “infectious foci with metacestode structure” according to the definition in our previous study [4]. Depending on the type of lesion, cellSens Dimension software (Olympus, Tokyo, Japan) was used to assess the areas of cell infiltration or granuloma in the entire section.

For Sirius red staining, a total of 3–5 fields/section/sample were quantified by computer using cellSens Dimension software, and the results were expressed as the percentage of positive staining per field.

For immunohistochemical staining, sections were subjected to heat-mediated antigen retrieval in Tris-ethylene diamine tetraacetic acid (EDTA) buffer. Subsequently, sections were blocked with 10% goat serum in phosphate-buffered saline (PBS) for 1 h at room temperature. Following blocking, sections were incubated with primary antibodies diluted in blocking solution at 4 °C overnight (anti-mouse alpha-smooth muscle actin [α-SMA], 1:500, ab124964). The next day, sections were washed with PBS and incubated with secondary antibody [goat anti-rabbit F(ab′)2-horseradish peroxidase (HRP)] at room temperature for 2 h. Staining was performed using a 3,3′diaminobenzidine (DAB) substrate kit (Abcam). A total of 3–5 areas/section/sample were then quantified by computer using cellSens Dimension software at ×100 magnification. The results were expressed as the intensity of positive staining per field.

### Adoptive transfer

Splenocytes were isolated from wild-type (CD45.1) and LAG3-KO (CD45.2) mice and mixed at a 1:1 ratio, and then 2 × 10^7^ total cells per mouse were co-transferred intravenously (i.v.) into CD4-KO recipient mice 1 day prior to infection with *E. multilocularis*. Recipients were euthanized 1 month after adoptive transfer, and mononuclear cells were isolated from the liver and spleen for subsequent analysis.

### Antibody treatment in vivo

Wild-type C57BL/6 mice were administered 200 μg of anti-LAG3 monoclonal antibody (mAb) (clone C9B7W, BioXCell) or an isotype control (Rat IgG1, κ, BioXCell) 2 days and 1 day prior to *E. multilocularis* infection via intraperitoneal injection (i.p.), followed by subsequent injections of 200 μg antibody or isotype control every 3 days thereafter.

### Mouse liver and spleen mononuclear cell isolation and flow cytometry

Single-cell suspensions of mononuclear cells were isolated from the liver and spleen as described previously [6]. For cell counting, 10 μl of the single-cell suspension was mixed with an equal volume of trypan blue, and 10 μl of the mixture was then loaded onto a hemocytometer. Viable cells were counted under an optical microscope. The absolute number of specific cell subsets (e.g., CD4^+^ T cells) was calculated by multiplying the total viable cell count by the proportion of target population determined via flow cytometry. A flowchart detailing the gating strategy for flow cytometry is provided as Additional file 1: Fig. S1.

For flow cytometric analysis, single-cell suspension was incubated in staining buffer (PBS containing 0.2% bovine serum albumin [BSA] and 0.1% sodium azide) with purified anti-CD16/CD32 at 4 °C for 30 min. Cell surface, intracellular cytokines, and nucleus staining were then performed according to established protocols [4, 5]. Samples were acquired on an LSRFortessa flow cytometer, and information regarding the antibodies used in this article is provided in Additional file 1: Table S1.

### Single-cell RNA sequencing dataset selection and processing

The processed public scRNA-seq dataset from *E. multilocularis*-infected mice was obtained from the National Center for Biotechnology Information (NCBI) database (GSE207480) [13]. Data analysis was performed using CellRanger software (v5.0; 10X Genomics). Uniform Manifold Approximation and Projection (UMAP) was applied for dimensionality reduction, followed by cell clustering and annotation. Detailed analytical procedures were described previously [14, 15].

### Statistical analysis

Flow cytometry data were analyzed using the FlowJo™ v10.6.1 program, and statistical analysis was performed using IBM SPSS Statistics 25; results were expressed as mean + standard error. Independent-sample *t*-tests were used to compare the two groups, and paired-samples *t*-tests or one-way analysis of variance (ANOVA) test was used to compare the intra-group data. For all experimental results, *P* < 0.05 was considered to be significant. (*P*-values are expressed as follows: *P < 0.05; **P < 0.01; ***P < 0.001).

## Results

### LAG3 deficiency contributes to early clearance of *E. multilocularis* infection

**Fig. 1:**
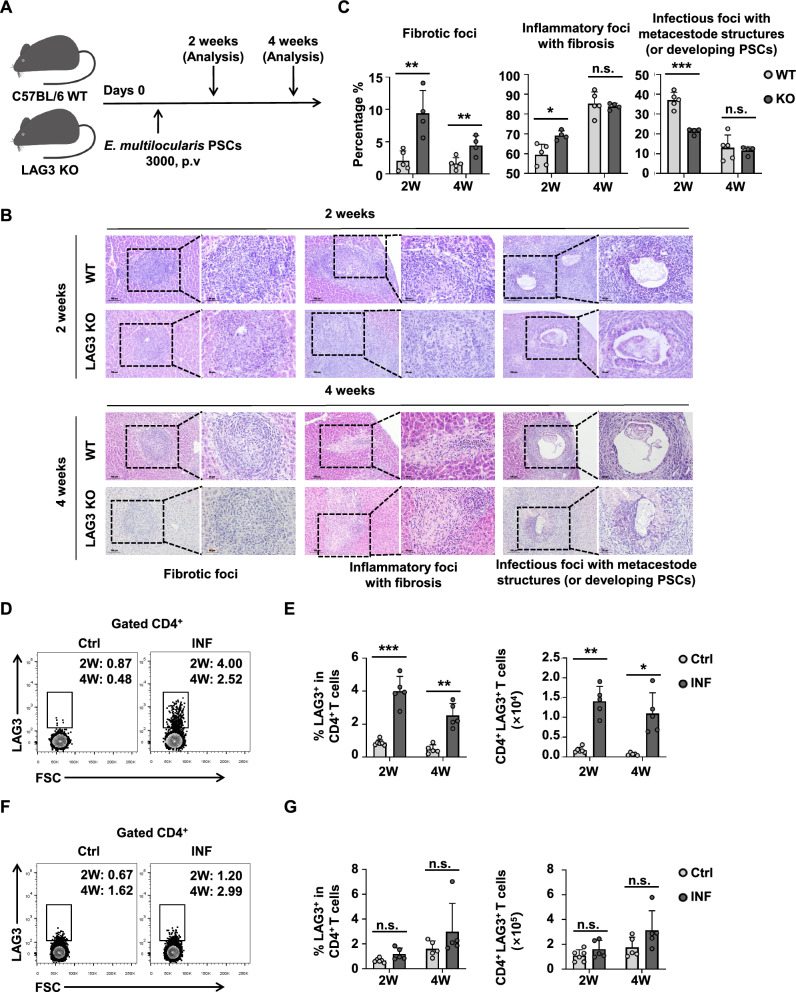
LAG3 expression is upregulated in liver CD4^+^ T cells, and its deficiency promotes *E. multilocularis* clearance at the early stages in infected mice. **A** Establishment of a protocol in the early stage of mouse *E. multilocularis* infection. **B** Representative hematoxylin–eosin staining image (left panel ×200, enlarged ×400 on the right panel) in the liver tissue sections from wild type (WT) and LAG3-KO mice after 2 and 4 weeks of infection. **C** Percentage of different lesion types in the liver from WT and LAG3-KO mice after 2 and 4 weeks of infection (4–5 mice per group). **D** Representative flow cytometry plot of LAG3 expression by CD4^+^ T cells in the liver from control (Ctrl) and *E. multilocularis*-infected (INF) mice after 2 and 4 weeks. **E** Percentage and absolute numbers of LAG3 expression by CD4^+^ T cells in the liver from Ctrl and INF mice after 2 and 4 weeks (5–6 mice per group). **F** Representative flow cytometry plot of LAG3 expression by CD4^+^ T cells in the spleen from Ctrl and INF mice after 2 and 4 weeks. **G** Percentage and absolute numbers of LAG3 expression by CD4^+^ T cells in the spleen from Ctrl and INF mice after 2 and 4 weeks (5–6 mice per group). All data are presented as mean + standard deviation (SD). *P < 0.05, **P < 0.01, ***P < 0.001, n.s., *P* > 0.05

To investigate the regulation of LAG3 on CD4^+^ T cells at early stages of infection, we established a murine model of early *E. multilocularis* infection (Fig. 1A, Additional file 1: Figs. S2A and 1B). The results showed that LAG3 deficiency significantly increased the proportion of fibrotic foci and inflammatory foci with fibrosis in the liver at 2 weeks post-infection (Fig. 1B and C). In contrast, the proportion of infectious foci with metacestode structures (protoscoleces developing into metacestode) decreased markedly in LAG3-deficient mice compared to WT mice during *E. multilocularis* establishment (Fig. 1B and C). However, LAG3 deficiency did not alter the expression levels of α-SMA across different lesion types (Additional file 1: Figs. S2C and 1D).

Previous studies from our group have shown that LAG3 is upregulated in CD4^+^ T cells during early *E. multilocularis* infection [4]. To further elucidate the changes in LAG3 expression in local versus peripheral immunity during early infection, we analyzed its expression on CD4^+^ T cells in the liver and spleen. The results showed that the proportion and number of CD4^+^LAG3^+^ T cells were significantly increased in the livers of *E. multilocularis*-infected mice (Fig. 1D and E), but there was no difference in the spleen (Fig. 1F and G). These findings suggest that *E. multilocularis* induces LAG3 expression in liver CD4^+^ T cells during early infection, thus facilitating immune evasion and supporting parasitic establishment.

### Upregulation of LAG3 expression on CD4^+^ Teff cells in the liver in a mouse model of early *E. multilocularis* infection

**Fig. 2:**
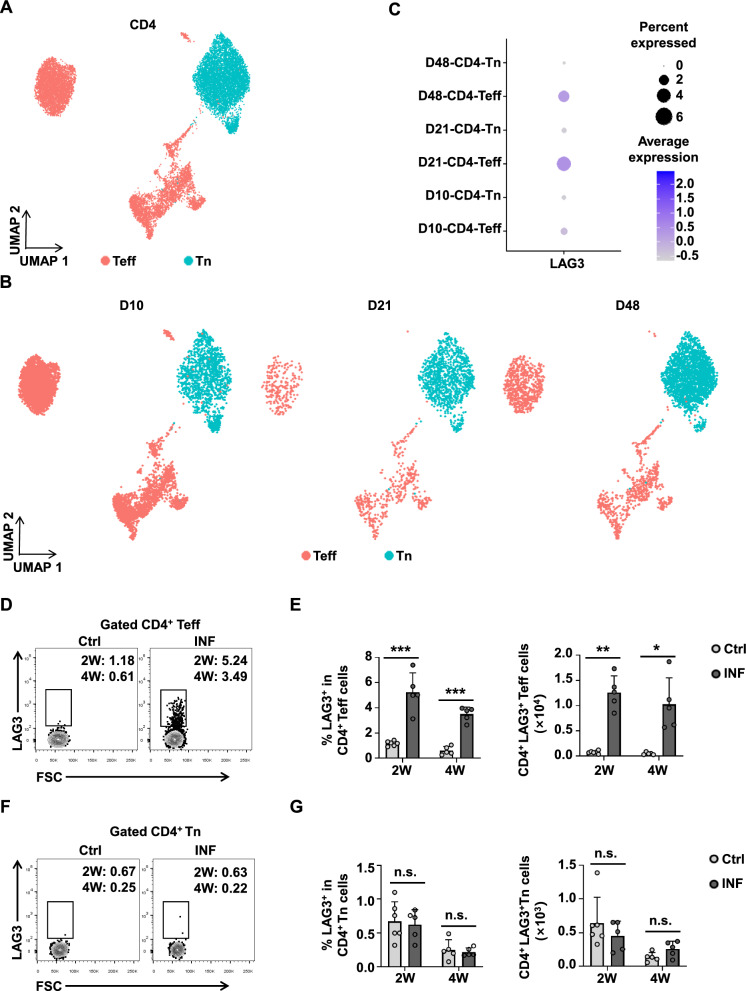
LAG3 expression is upregulated on liver CD4^+^ Teff cells at the early stages in *E. multilocularis*-infected mice. **A** Uniform Manifold Approximation and Projection (UMAP) plot showing the annotation and color codes for CD4^+^ T cell types. **B** UMAP plot showing the origin of CD4^+^ T cell types at different times of infection. **C** The bubble chart shows the expression levels of LAG3 on CD4^+^ Teff cells (CD4^+^CD44^+^CD62L^−^) and CD4^+^ Tn cells (CD4^+^CD44^−^CD62L^+^) at different times of infection. **D** Representative flow cytometry plot of LAG3 expression in CD4^+^ Teff cells in the liver from Ctrl and INF mice after 2 and 4 weeks. **E** Percentage and absolute numbers of LAG3 expression in CD4^+^ Teff cells in the liver from Ctrl and INF mice after 2 and 4 weeks (5–6 mice per group). **F** Representative flow cytometry plot of LAG3 expression in CD4^+^ Tn cells in the liver from Ctrl and INF mice after 2 and 4 weeks. **G** Percentage and absolute numbers of LAG3 expression in CD4^+^ Tn cells in the liver from Ctrl and INF mice after 2 and 4 weeks (5–6 mice per group). All data are presented as mean + SD. *P < 0.05, **P < 0.01, ***P < 0.001, n.s., *P* > 0.05

To characterize the distribution of LAG3 expression across CD4^+^ T cell subsets, we obtained and analyzed a publicly available single-cell sequencing dataset from mice with early *E. multilocularis* infection (GSE207480). CD4^+^ T cells were clustered and divided into two major subsets: naive CD4^+^ T cells (CD4^+^ Tn, CD4^+^CD44^−^CD62L^+^) and CD4^+^ Teff cells (CD4^+^CD44^+^CD62L^−^) (Fig. 2A). Subsequently, we showed the distribution of CD4^+^ T cell subsets at days 10, 21, and 48 post-infection (Fig. 2B). Analysis of LAG3 expression revealed that it was predominantly expressed within the CD4^+^ Teff cell population (Fig. 2C).

To validate these findings, we further performed flow cytometry and confirmed that LAG3 expression was significantly upregulated on CD4^+^ Teff cells, whereas no notable change was observed in CD4^+^ Tn cells in the livers of mice infected with *E. multilocularis* (Fig. 2D–G). In the spleen, a slight increase in LAG3 expression was detected across CD4^+^ T cell subsets following infection, although the differences were not statistically significant (Additional file 1: Fig. S3A–D). To further evaluate the effect of LAG3 on CD4^+^ T cell differentiation, we utilized LAG3-deficient mice. Interestingly, LAG3 deficiency did not affect the differentiation of CD4^+^ T cell subsets in the liver during early infection (Additional file 1: Fig. S4A–C). In the spleen, however, although the proportion and number of CD4^+^ T cells were reduced, LAG3 deficiency promoted the differentiation of CD4^+^ Tn cells into CD4^+^ Teff cells (Additional file 1: Fig. S4D–F). Collectively, LAG3 is upregulated on CD4^+^ Teff cells in the liver during the early stages of *E. multilocularis* infection and plays a subset-specific regulatory role in peripheral T cell differentiation.

### LAG3 is mainly upregulated on CD4^+^CD44^+^CD69^+^ T cells in the liver in a mouse model of early *E. multilocularis* infection

**Fig. 3:**
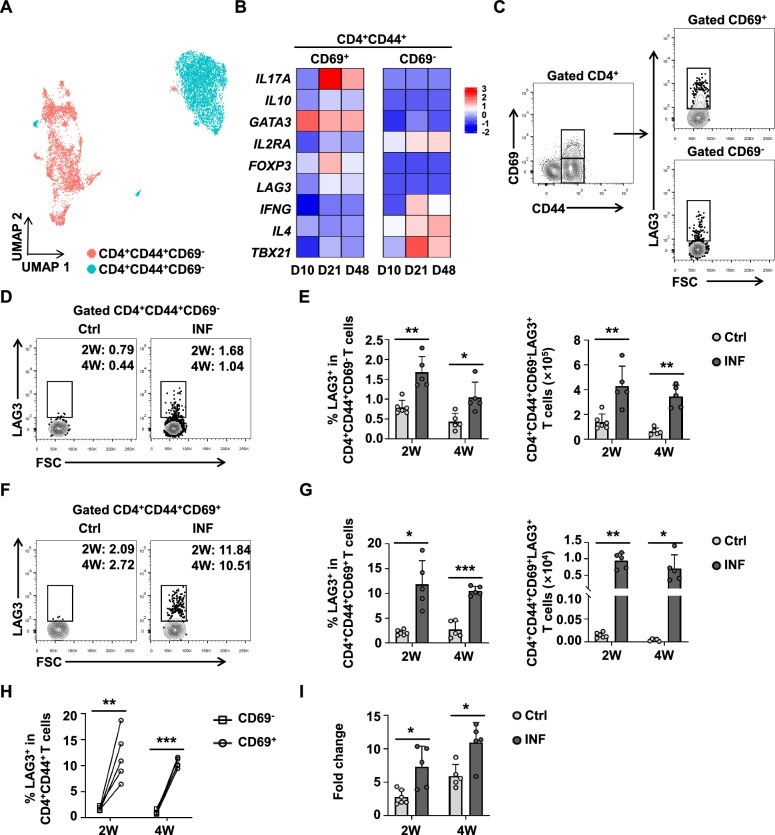
LAG3 is mainly expressed on liver CD4^+^CD44^+^CD69^+^ T cells at the early stages of *E. multilocularis*-infected mice. **A** UMAP plot showing the annotations and color codes for CD4^+^CD44^+^CD69^+^ and CD4^+^CD44^+^CD69^−^ T cells. **B** Heatmap indicating the expression of selected genes in CD4^+^CD44^+^CD69^+^ and CD4^+^CD44^+^CD69^−^ T cell subtypes. **C** Two-dimensional scatterplot showing the gating strategy used to distinguish between liver CD4^+^CD44^+^CD69^−^ and CD4^+^CD44^+^CD69^+^ T cells. **D** Representative flow cytometry plot of LAG3 expression by CD4^+^CD44^+^CD69^−^ T cells in liver from Ctrl and INF mice after 2 and 4 weeks. **E** Percentage and absolute numbers of LAG3 expression by CD4^+^CD44^+^CD69^−^ T cells in liver from Ctrl and INF mice after 2 and 4 weeks (5–6 mice per group). **F** Representative flow cytometry plot of LAG3 expression by CD4^+^CD44^+^CD69^+^ T cells in the liver from Ctrl and INF mice after 2 and 4 weeks. **G** Percentage and absolute numbers of LAG3 expression by CD4^+^CD44^+^CD69^+^ T cells in the liver from Ctrl and INF mice after 2 and 4 weeks (5–6 mice per group). **H** Percentage of LAG3 expression by CD4^+^CD44^+^CD69^−^ and CD4^+^CD44^+^CD69^+^ T cells in the liver from INF mice after 2 and 4 weeks (five mice per group). **I** The ratio of LAG3 expression on CD4^+^CD44^+^CD69^+^ and CD4^+^CD44^+^CD69^−^ T cells. All data are presented as mean + SD. *P < 0.05, **P < 0.01, ***P < 0.001

To further investigate LAG3 expression within CD4^+^ Teff, we subdivided this population into CD4^+^CD44^+^CD69^+^ and CD4^+^CD44^+^CD69^−^ T cell subsets (Fig. 3A). Transcriptional analysis indicated higher LAG3 mRNA level in the CD4^+^CD44^+^CD69^+^ T cell subsets (Fig. 3B). We subsequently validated these findings using flow cytometry (Fig. 3C and Additional file 1: Fig. S5A). The proportion and number of LAG3 expression in CD4^+^CD44^+^CD69^−^ and CD4^+^CD44^+^CD69^+^ T cells in the liver of infected mice were higher than that of the uninfected mice at the early stages (Fig. 3D–G). Notably, the proportion of LAG3 expressed by CD4^+^CD44^+^CD69^+^ T cells in liver was significantly higher than that of CD4^+^CD44^+^CD69^−^ T cells (Fig. 3H). Further comparative analysis confirmed that the extent of LAG3 upregulation following infection was more pronounced in CD4^+^CD44^+^CD69^+^ T cells relative to their CD69^−^ counterparts (Fig. 3I). In the spleen, although LAG3 expression was higher on CD4^+^CD44^+^CD69^+^ T cells than on CD4^+^CD44^+^CD69^−^ T cells, no differences were observed between infected and uninfected mice (Additional file 1: Fig. S5B–F). Collectively, these results suggest that LAG3 expression is mainly upregulated on liver CD4^+^CD44^+^CD69^+^ T cells following *E. multilocularis* infection.

### LAG3-expressing CD4^+^CD44^+^ T cells secrete more IL-4 and IL-10 in a mouse model of early *E. multilocularis* infection

**Fig. 4:**
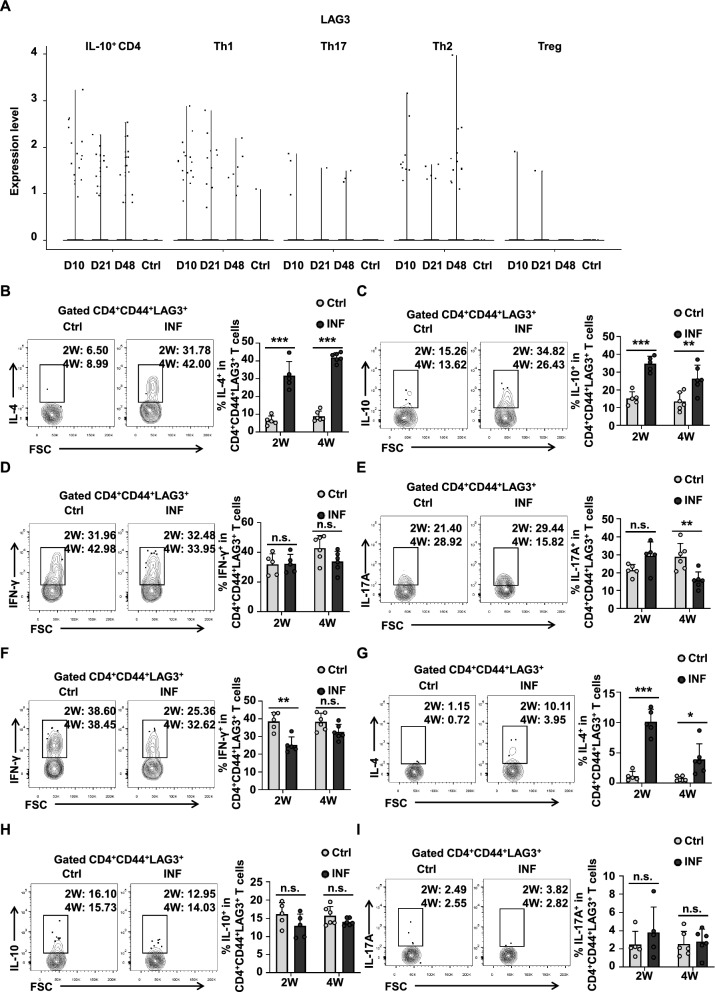
LAG3-expressing CD4^+^CD44^+^ T cells secrete more IL-4 and IL-10 from the liver at the early stages of *E. multilocularis*-infected mice. **A** Violin diagram shows the expression of LAG3 in CD4^+^ T cell subsets in the liver of uninfected (Ctrl) and *E. multilocularis*-infected (INF) mice at different times. **B** Representative flow cytometry plot and percentage of IL-4 production by CD4^+^CD44^+^LAG3^+^ T cells in the liver from Ctrl and INF mice after 2 and 4 weeks of infection (5–6 mice per group). **C** Representative flow cytometry plot and percentage of IL-10 production by CD4^+^CD44^+^LAG3^+^ T cells in the liver from Ctrl and INF mice after 2 and 4 weeks of infection (5–6 mice per group). **D** Representative flow cytometry plot and percentage of IFN-γ production by CD4^+^CD44^+^LAG3^+^ T cells in the liver from Ctrl and INF mice after 2 and 4 weeks of infection (5–6 mice per group). **E** Representative flow cytometry plot and percentage of IL-17A production by CD4^+^CD44^+^LAG3^+^ T cells in the liver from Ctrl and INF mice after 2 and 4 weeks of infection (5–6 mice per group). **F** Representative flow cytometry plot and percentage of IFN-γ production by CD4^+^CD44^+^LAG3^+^ T cells in the spleen from Ctrl and INF mice after 2 and 4 weeks of infection (5–6 mice per group). **G** Representative flow cytometry plot and percentage of IL-10 production by CD4^+^CD44^+^LAG3^+^ T cells in the spleen from Ctrl and INF mice after 2 and 4 weeks of infection (5–6 mice per group). **H** Representative flow cytometry plot and percentage of IL-4 production by CD4^+^CD44^+^LAG3^+^ T cells in the spleen from Ctrl and INF mice after 2 and 4 weeks of infection (5–6 mice per group). **I** Representative flow cytometry plot and percentage of IL-17A production by CD4^+^CD44^+^LAG3^+^ T cells in the spleen from Ctrl and INF mice after 2 and 4 weeks of infection (5–6 mice per group). All data are presented as mean + SD. **P < 0.01, ***P < 0.001, n.s., *P* > 0.05

To elucidate LAG3 expression during early *E. multilocularis* infection, we reanalyzed RNA-seq data and observed upregulation of LAG3 across multiple CD4^+^ T cell subsets following *E. multilocularis* infection (Fig. 4A). In addition, higher expression levels of LAG3 were detected in IL10-producing CD4^+^ T cells and Th2 cell populations (Fig. 4A). To validate these findings, we assessed cytokine production in CD4^+^CD44^+^LAG3^+^ T cells by flow cytometry. In the liver, CD4^+^CD44^+^LAG3^+^ T cells from infected mice secreted significantly higher levels of IL-4 and IL-10 compared to those from uninfected mice at both 2 and 4 weeks (Fig. 4B and C). In contrast, IFN-γ production remained unchanged (Fig. 4D), while IL-17A secretion was significantly reduced in CD4^+^CD44^+^LAG3^+^ T cells from infected mice at 4 weeks (Fig. 4E). In the spleen, CD4^+^CD44^+^LAG3^+^ T cells from infected mice produced more IL-4 but less IFN-γ than those from uninfected mice at both 2 and 4 weeks (Fig. 4F and G). However, no significant differences were observed in IL-10 or IL-17A production between infected mice and uninfected mice (Fig. 4H and I). Collectively, these results indicate that LAG3 expression was enriched in memory Th2 and Treg subsets in both the liver and spleen during early infection.

### LAG3 deficiency enhances the secretion of more IFN-γ, IL-4, and IL-10 by splenic CD4^+^ T cells in a mouse model of early *E. multilocularis* infection

**Fig. 5:**
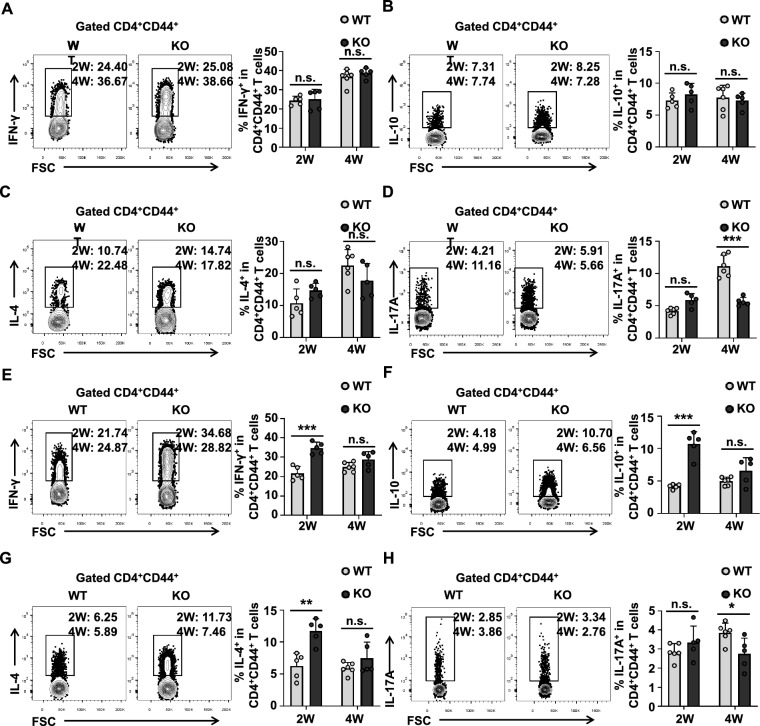
LAG3 deficiency promotes the secretion of more IFN-γ, IL-4, and IL-10 by splenic CD4^+^CD44^+^ T cell at the early stages of *E. multilocularis*-infected mice. **A** Representative flow cytometry plot and percentage of IFN-γ production by CD4^+^CD44^+^ T cells in the liver from WT and LAG3-KO mice after 2 and 4 weeks of infection (5–6 mice per group). **B** Representative flow cytometry plot and percentage of IL-10 production by CD4^+^CD44^+^ T cells in the liver from WT and LAG3-KO mice after 2 and 4 weeks of infection (5–6 mice per group). **C** Representative flow cytometry plot and percentage of IL-4 production by CD4^+^CD44^+^ T cells in the liver from WT and LAG3-KO mice after 2 and 4 weeks of infection (5–6 mice per group). **D** Representative flow cytometry plot and percentage of IL-17A production by CD4^+^CD44^+^ T cells in the liver from WT and LAG3-KO mice after 2 and 4 weeks of infection (5–6 mice per group). **E** Representative flow cytometry plot and percentage of IFN-γ production by CD4^+^CD44^+^ T cells in the spleen from WT and LAG3-KO mice after 2 and 4 weeks of infection (5–6 mice per group). **F** Representative flow cytometry plot and percentage of IL-10 production by CD4^+^CD44^+^ T cells in the spleen from WT and LAG3-KO mice after 2 and 4 weeks of infection (5–6 mice per group). **G** Representative flow cytometry plot and percentage of IL-4 production by CD4^+^CD44^+^ T cells in the spleen from WT and LAG3-KO mice after 2 and 4 weeks of infection (5–6 mice per group). **H** Representative flow cytometry plot and percentage of IL-17A production by CD4^+^CD44^+^ T cells in the spleen from WT and LAG3-KO mice after 2 and 4 weeks of infection (5–6 mice per group). All data are presented as mean + SD. *P < 0.05, **P < 0.01, ***P < 0.001, n.s., *P* > 0.05

To further investigate the functional impact of LAG3 on CD4^+^ T cells during early *E. multilocularis* infection, we infected LAG3-deficient mice with *E. multilocularis*. LAG3 deficiency specifically suppressed Th17 cell differentiation at 4 weeks post-infection (Fig. 5A–D), but did not significantly affect the formation of Treg cells (Additional file 1: Fig. S6A and B). Furthermore, LAG3 deficiency promoted the proliferation of Tconv and Treg cells at 2 weeks but inhibited the Treg proliferation at 4 weeks (Additional file 1: Fig. S6C and D). Interestingly, although no significant difference in LAG3 expression was observed in the spleens between infected and uninfected mice (Fig. 1F and G), LAG3 deficiency promoted the production of IFN-γ, IL-4, and IL-10 by splenic CD4^+^CD44^+^ T cells during early infection and reduced the Treg population (Fig. 5E–H and Additional file 1: Fig. S6E and F). In contrast, LAG3 deficiency did not affect the proliferation of Tconv or Treg cells in the spleen (Additional file 1: Fig. S6G and H).

**Fig. 6:**
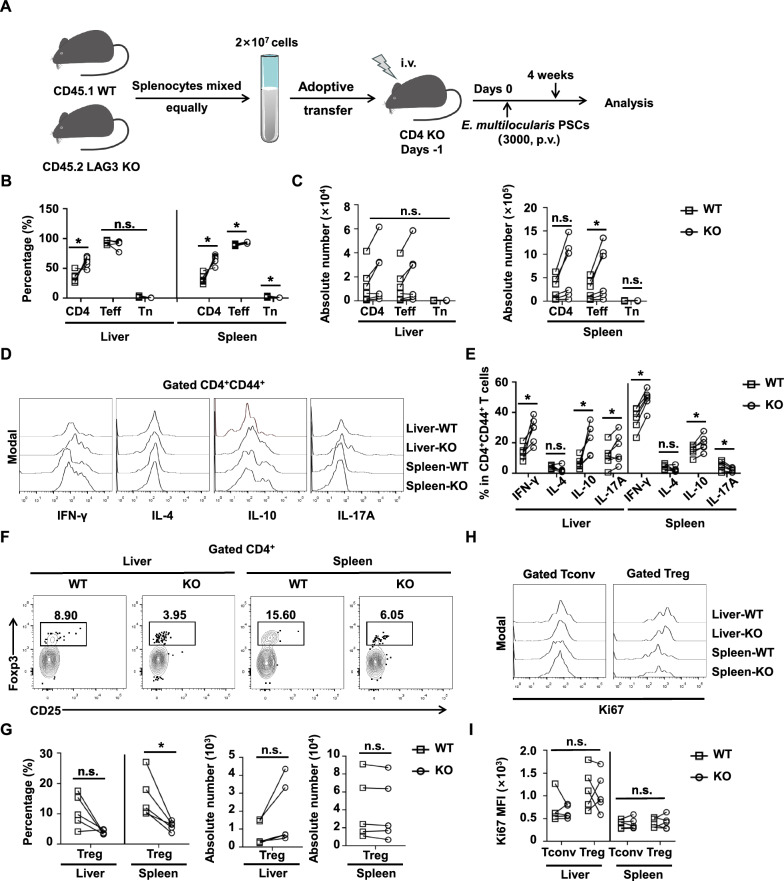
LAG3-deficient CD4^+^ T cell secreted more IFN-γ and IL-10 upon adoptive transfer into CD4-KO mice and subsequent *E. multilocularis* infection. **A** Establishment of adoptive transfer mouse model. **B** Percentage of transferred CD4^+^ T cells, CD4^+^ Tn, and CD4^+^ Teff in the liver and spleen of *E. multilocularis*-infected CD4-KO mice that received adoptive transfer (five mice per group). **C** Absolute number of transferred CD4^+^ T cells, CD4^+^ Tn, and CD4^+^ Teff in the liver and spleen of *E. multilocularis*-infected CD4-KO mice that received adoptive transfer (five mice per group). **D** Representative flow cytometry plot of IFN-γ, IL-4, IL-10, and IL-17A production by transferred CD4^+^CD44^+^ T cells in the liver and spleen from *E. multilocularis*-infected CD4-KO mice. **E** Percentage of IFN-γ, IL-4, IL-10, and IL-17A production by transferred CD4^+^CD44^+^ T cells in the liver and spleen from *E. multilocularis*-infected CD4-KO mice (five mice per group). **F** Representative flow cytometry plot of Treg cells in the liver and spleen from *E. multilocularis*-infected CD4-KO mice that received adoptive transfer. **G** Percentage and absolute number of Treg cells in the liver and spleen from *E. multilocularis*-infected CD4-KO mice that received adoptive transfer (five mice per group). **H** Representative flow cytometry plot of Ki67 expression in Tconv and Treg cells in the liver and spleen from *E. multilocularis*-infected CD4-KO mice that received adoptive transfer. **I** Median fluorescence intensity (MFI) of Ki67 expression in Tconv and Treg cells in the liver and spleen from *E. multilocularis*-infected CD4-KO mice that received adoptive transfer (five mice per group). All data are presented as mean + SD. *P < 0.05, n.s., *P* > 0.05

To more precisely define the role of LAG3 in early infection, we established an adoptive transfer model of CD4-KO mice recipients 1 day before *E. multilocularis* infection (Fig. 6A). Both the proportions and absolute numbers of CD4^+^ T cells and CD4^+^ Teff cells were higher in LAG3-KO donor cells than WT donor cells in the liver and spleen, although some differences were not statistically significant (Fig. 6B and C). The levels of IFN-γ and IL-10 in CD4^+^ T cells from LAG3-KO donors were significantly upregulated compared with that of WT donors (Fig. 6D and E). Additionally, the level of IL-17 was significantly increased in liver CD4^+^ T cells from LAG3-KO donors compared with that of WT donors but decreased in splenic cells (Fig. 6D and E). The CD4^+^ T cells of LAG3-KO donors had similar expression of IL-4 as WT donors (Fig. 6D and E). The proportion of Treg was lower in liver and spleen cells from LAG3-KO donor cells than that from WT donor cells, but there was no statistically significant difference in absolute Treg number or Ki67 expression (Fig. 6F–I). Overall, these results indicated that LAG3 deficiency may enhance antiparasitic immunity by promoting the Th1 response in the peripheral spleen, though it simultaneously amplifies Th2 cytokine production.

### LAG3 blockade significantly increases the area of inflammatory foci with fibrosis while only mildly inhibiting the progression of metacestode lesions in a mouse model of early *E. multilocularis* infection

**Fig. 7:**
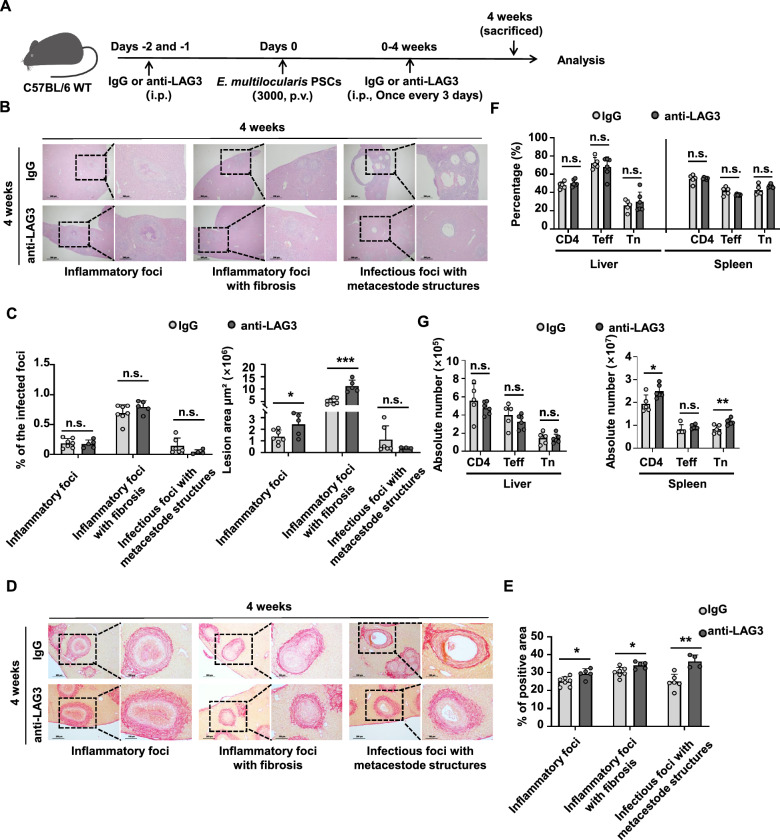
LAG3 blockade promotes early clearance of *E. multilocularis* in mouse liver. **A** Establishment of a LAG3-neutralizing antibody model in vivo. **B** Representative hematoxylin–eosin staining image (left panel ×40, enlarged ×100 on the right panel) in the liver tissue sections of *E. multilocularis*-infected mice treated with IgG and anti-LAG3 mAb for 4 weeks. **C** Percentage and area of different lesion types in the liver of *E. multilocularis*-infected mice treated with IgG (*n* = 7) and anti-LAG3 mAb (*n* = 5) for 4 weeks. **D** Representative Sirius red staining image (left panel ×100, enlarged ×200 on the right panel) in the liver tissue sections of *E. multilocularis*-infected mice treated with IgG and anti-LAG3 mAb for 4 weeks. **E** Percentage of different lesion types in the liver of *E. multilocularis*-infected mice treated with IgG (*n* = 7) and anti-LAG3 mAb (*n* = 5) for 4 weeks. **F** Percentage of CD4^+^ T cells, CD4^+^ Tn, and CD4^+^ Teff in the liver and spleen of *E. multilocularis*-infected mice treated with IgG (*n* = 5) and anti-LAG3 mAb (*n* = 6) for 4 weeks. **G** Absolute number of CD4^+^ T cells, CD4^+^ Tn, and CD4^+^ Teff in the liver and spleen of *E. multilocularis*-infected mice treated with IgG (*n* = 5) and anti-LAG3 mAb (*n* = 6) for 4 weeks. All data are presented as mean + SD. *P < 0.05, **P < 0.01, ***P < 0.001, n.s., *P* > 0.05

We further treated *E. multilocularis*-infected mice with anti-LAG3 mAb or IgG during early infection (Fig. 7A). Pathological examination revealed that LAG3 blockade significantly increased the area of inflammatory foci and inflammatory foci with fibrosis (Fig. 7B and C). Furthermore, although not statistically significant, a trend toward reduction in the area of infectious foci with metacestode structures was observed in the livers of anti-LAG3-treated mice (Fig. 7B and C). In addition, Sirius red staining showed that the degree of fibrosis across various lesion types was significantly enhanced in the liver following anti-LAG3-treatment (Fig. 7D and E).

We next assessed the effect of LAG3 blockade on CD4^+^ T cell phenotype and functions by flow cytometry. While no significant difference was detected in the percentage of CD4^+^ T cell subsets in either the liver or spleen between anti-LAG3-treated mice and the IgG group, the absolute number of splenic CD4^+^ T cells and CD4^+^ Tn cells was increased in anti-LAG3-treated mice (Fig. 7F and G). The percentage of IL-4-producing CD4^+^CD44^+^ T cells was increased in the liver, while the percentage of IL-17A decreased in anti-LAG3-treated mice (Additional file 1: Fig. S7A and B). Moreover, the percentage of IL-10-producing CD4^+^CD44^+^ T cells was significantly increased in the liver and spleen of anti-LAG3-treated mice, whereas IFN-γ production remained unchanged (Additional file 1: Fig. S7A and B). The proportion of Treg cells was unaltered in the liver and spleen (Additional file 1: Fig. S7C and D). However, Ki67 expression was increased in liver Tconv cells and decreased in spleen Treg cells in anti-LAG3-treated mice (Additional file 1: Fig. S7E and F). Collectively, these findings indicate that anti-LAG3 treatment modestly impedes the progression of hepatic parasitic lesions during the early stages of *E. multilocularis* infection, associated with modulated CD4^+^ T cell cytokine responses and altered fibrotic pathology.

## Discussion

As pivotal regulators of adaptive immune, CD4^+^ T cells play a complex dual role in anti-parasitic infections [5, 6, 16, 17]. In *E. multilocularis* infection models, CD4^+^ T cells depletion accelerates metacestode proliferation, underscoring their protective function [5]. Further studies reveal functional heterogeneity within the CD4^+^ T cell population: CD4^+^IFN-γ^+^ effector T cells mediate direct parasite killing via macrophage activation, whereas CD4^+^ IL-10^+^ Treg cells promote parasite immune escape by suppressing immune responses [6, 16, 17]. This delicate balance between pro- and anti-inflammatory forces ultimately determines infection outcome [6, 16, 17]. Building upon our prior finding that LAG3 deficiency significantly reduces parasitic burden in chronic *E. multilocularis* infection, this study elucidates its critical role in reshaping the immune microenvironment during early infection, offering novel insights for overcoming current therapeutic challenges in AE. Here, we discovered that LAG3 was mainly present on CD4^+^ Teff cells and further confirmed that LAG3 deficiency remodels the peripheral immune microenvironment, activating splenic CD4^+^ Teff cells to mediated immune clearance of *E. multilocularis* in early infection. This occurs largely through modulation of Th1 and Th2 response by splenic CD4^+^ T cells. Notably, the anti-parasitic effect of LAG3 deficiency was most pronounced at 2 weeks post-infection and diminished by 4 weeks, suggesting involvement of additional regulatory molecules during this period and highlighting the time-sensitive nature of intervention strategies.

CD4^+^ Teff cells constitute a persistent defense line against infection, with newly differentiated subsets exhibiting specific recognition of parasite antigens post-infection [4, 18]. LAG3, an immune checkpoint molecule expressed on both CD4^+^ Tn and CD4^+^ Teff cells, regulates T cell differentiation trajectories [19, 20]. Given that liver tissue-resident T cells exert prolonged local immunomodulatory effects, we focused particularly on LAG3 expression in this population. Our study reveals critical spatio-temporal heterogeneity: (i) With regard to organ-specific regulation, while LAG3 is constitutively expressed on CD4^+^ Tn and CD4^+^ Teff cells in both liver and spleen, significant infection-induced upregulation occurs only on hepatic CD4^+^ Teff cells. (ii) Regarding the liver microenvironment polarization, early infection synchronously increases LAG3 expression on both CD4^+^CD44^+^CD69⁻ (circulating) and CD4^+^CD44^+^CD69^+^ (tissue-resident) T cells in the liver, with the greatest upregulation in CD69^+^ cells. This dual heterogeneity (organ- and subset-specific) indicates that *E. multilocularis* reshapes the hepatic immune microenvironment upon invasion, establishing an immunosuppressive niche via LAG3 upregulation [21, 22]. Although LAG3 deficiency did not alter the CD4^+^ Tn → Teff conversion efficiency in the liver, it significantly promoted splenic CD4^+^ Teff differentiation. Coupled with evidence of efficient protoscoleces (PSCs) killing by splenocytes in vitro [23], we propose a new paradigm of “peripheral immune priming-hepatic effector execution,” wherein LAG3 deletion activates splenic CD4^+^ Teff cells, disrupting the early immunosuppressive barrier established by the parasite.

IFN-γ serves as a key cytokine for parasite clearance, while IL-10 promotes parasite survival via immunosuppression [24–28]. LAG3 deficiency induced a unique pattern in splenic CD4^+^CD44^+^ T cells, co-upregulating both IFN-γ and IL-10. This seemingly paradoxical response transfers to the liver during mid-infection [6], suggesting a cross-organ immunoregulatory compensatory mechanism. This study and previous research revealed that *E. multilocularis* can also influence its post-infection establishment by modulating peripheral immunity [23, 29–31]. Moreover, adoptive transfer experiments showed that LAG3-deficient splenic CD4^+^ T cells elevate both IFN-γ and IL-10 in CD4^+^CD44^+^ T cells of *E. multilocularis*-infected mice, demonstrating that peripheral immunity continuously modulates liver-localized responses. IL-10 likely originates from “dual-functional T cells” generated during Th1 cell differentiation—co-expressing IFN-γ/IL-10 and high levels of immune checkpoints, including PD-1, LAG3, and TIGIT [26, 32]. The limited efficacy of PD-1 blockade in *E. multilocularis* infection, associated with granulocytic myeloid-derived suppressor cell (G-MDSC) accumulation in the liver [33], underscores the parasite’s use of a multi-checkpoint strategy to ensure persistent infection.

IL-4, a hallmark Th2 cytokine, exhibits complex bidirectional regulatory in AE infection. It can suppress excessive inflammation to limit tissue damage [34], yet also promote fibrotic encapsulation for lesion isolation [35]. In immune responses, it recruits eosinophils to enhance parasite clearance [36, 37], while driving M2 macrophage polarization to form a pro-parasitic microenvironment [25]; its effect on the parasite itself is even more paradoxical—promoting early larval clearance [38], while potentially increasing transmission risk in chronic infection [34]. This study found that IL-4 secretion was significantly higher in liver CD4^+^CD44^+^LAG3^+^ T cells compared to uninfected controls during acute infection (2 and 4 weeks). Notably, LAG3 deficiency further enhanced IL-4 production in splenic CD4^+^CD44^+^ T cells, indicating IL-4 dynamics are driven more by infection stage and local inflammation than LAG3 expression. Temporal analysis suggests that early IL-4 elevation (0–2 weeks) confers host protection by recruiting eosinophils to direct larvae killing [36, 37], whereas sustained IL-4 expression beyond 12 weeks correlates with pathological fibrosis that may impede drug penetration [35]. This time-dependent role was reinforced in an adoptive transfer model: LAG3-deficient CD4^+^ T cells from donors suppressed IL-4 production when transferred to late infection recipients [6], but did affect IL-4 levels in recipients with early infection. Integrating multiple studies [35, 38, 39], we propose an “IL-4 immune clock” model: early IL-4 induction (0–4 weeks) constitutes a protective host response, activating eosinophil-mediated larval killing [36, 37], whereas mid–late-stage (> 12 weeks) IL-4 promotes parasite survival, highlighting the need for temporally precise targeting.

Traditionally, LAG3 transmits inhibitory signals by binding MHC-II [40], but new evidence shows it possesses immune-stimulatory capacity: soluble LAG3 can activate dendritic cells (DCs) to enhance T cell responses [41–45]. Using LAG3 neutralizing antibody (C9B7W) treatment, we found (i) superficial efficacy similarity, where both LAG3 genetic deletion and antibody blockade enhance parasite clearance; and (ii) mechanistic essential difference, where LAG3-KO increased IFN-γ production in CD4^+^CD44^+^ T cells, whereas antibody blockade did not alter IFN-γ levels. This discrepancy likely stems from LAG3’s dual mechanism: (i) the MHC II-dependent pathway (dominant), in which LAG3–MHC II binding recruits SHP-1 to inhibit T cell receptor (TCR) signaling [46, 47]; (ii) MHC II-independent pathway, with additional ligands (e.g., FGL1, galectin-3, LSECtin, α-synuclein) to engage LAG3 and dampen immune surveillance. Moreover, the LAG3 antibody (C9B7W) has been reported not to block LAG3-MHC Class II interaction but disrupts LAG3 dimerization and binding to the TCR/CD3 complex [48, 49]. This unique mechanism prevents it from completely mimicking the genetic deletion.

## Conclusions

This study demonstrates that LAG3 facilitates the early establishment of *E. multilocularis* infection by mediating immunosuppression. The co-upregulation of IFN-γ and IL4/IL-10 following in vivo LAG3 deficiency indicates the activation of a compensatory immunoregulatory network. Notably, both genetic deficiency and neutralizing antibody blockade of LAG3 enhance clearance of early *E. multilocularis* infection yet elicit divergent CD4^+^ T cell cytokine signatures (e.g., IFN-γ level). These findings provide a rational for shifting therapeutic strategies in AE from single-target inhibition toward dynamic network intervention.

## Supplementary Information

Additional file 1.

Additional file 2.

## Data Availability

All study data are included in the article and/or Supplementary Information.

## Abbreviations

AE: Alveolar echinococcosis
*E. multilocularis*: *Echinococcus multilocularis*
IFN-γ: Interferon-γ
IL-10: Interleukin-10
IL-17A: Interleukin-17A
IL-4: Interleukin-4
IL-13: Interleukin-13
KO: Knockout
LAG3: Lymphocyte activation gene-3
mAb: Monoclonal antibody
PD-1: Programmed death 1
PSCs: Protoscoleces
Teff: Effector T cell
Th1: T helper type 1
Th2: T helper type 2
Tn: Naïve T cell
TIGIT: T cell immunoreceptor with immunoglobulin [Ig] and immunoreceptor tyrosine-based inhibitory motif [ITIM] domain
Treg: Regulatory T cell
