# Supplementary material for: LAG3 constrains anti-parasitic response by effector CD4+ T-cell in early Echinococcus multilocularis-infected mice
Source: Parasit Vectors. 2026 Feb 12;19:122. doi: 10.1186/s13071-026-07246-y (PMC12998186; doi:10.1186/s13071-026-07246-y)
Supplement: Supplementary file 1 — Additional file 1. [file 13071_2026_7246_MOESM1_ESM.docx]

**
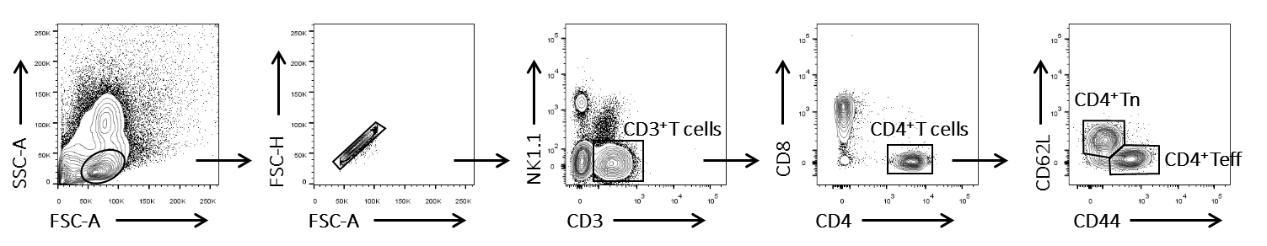
**

**Additional file 1: Fig. S 1 Representative flow cytometry plots showing the gating strategy for the flow cytometric analysis and identification of CD4^+^ T cell populations in *E. multilocularis* infected mice.** Tn: naïve T cell; Teff: effector T cell.


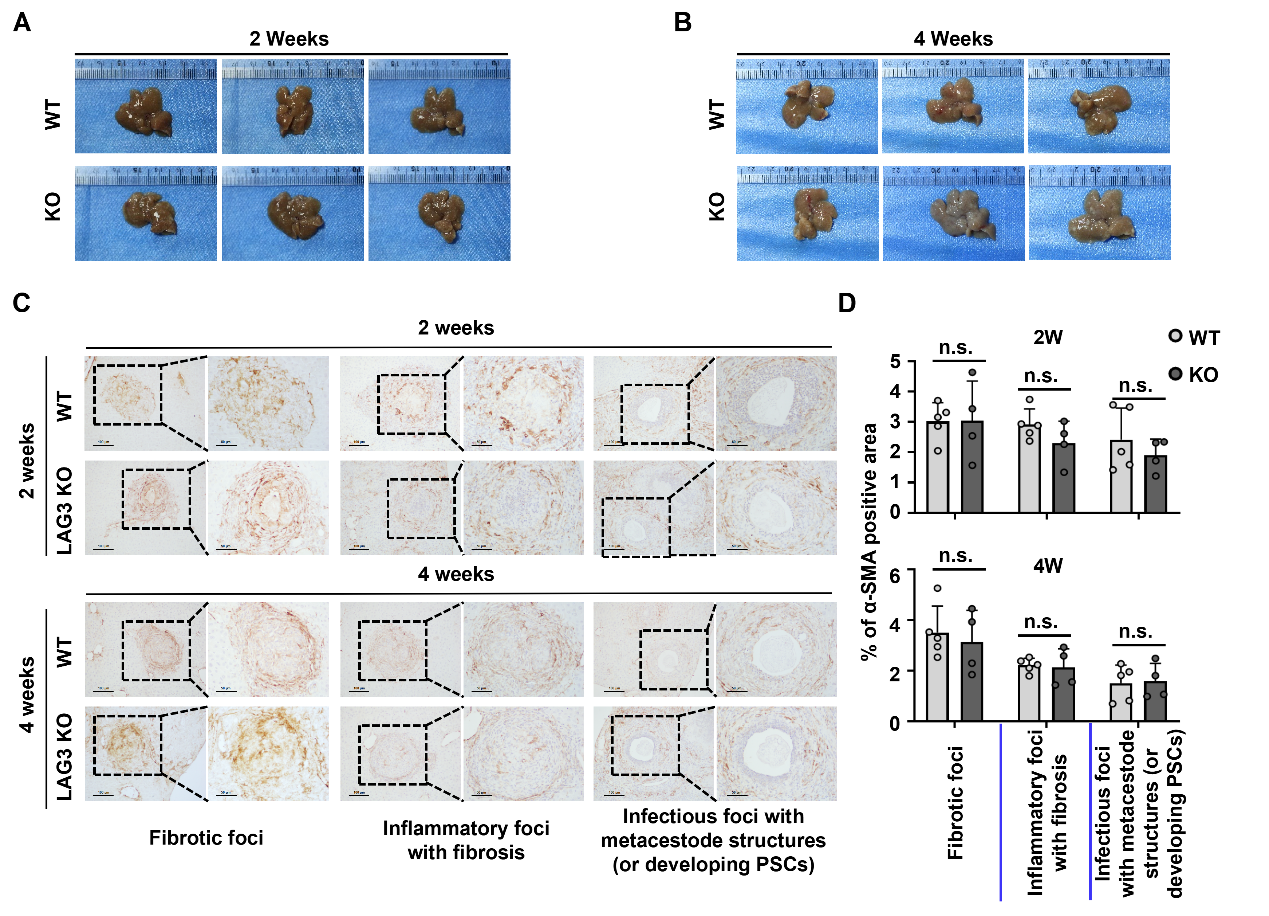


**Additional file 1: Fig. S 2 LAG3 did not affect the expression of α-SMA in the liver at the early stages of *E. multilocularis* infected mice.**
(A) Representative images of liver from WT and LAG3-KO mice after 2 weeks of infection. (B) Representative images of liver from WT and LAG3-KO mice after 4 weeks of infection. (C) Representative immunohistochemical staining of α-SMA (left panel 200×, enlarged 400× on the right panel) in the liver tissue sections from WT and LAG3-KO mice at 2 and 4 weeks of infection. (D) The percentage of positive staining area was calculated to evaluate the expression of α-SMA (4-5 mice per group).


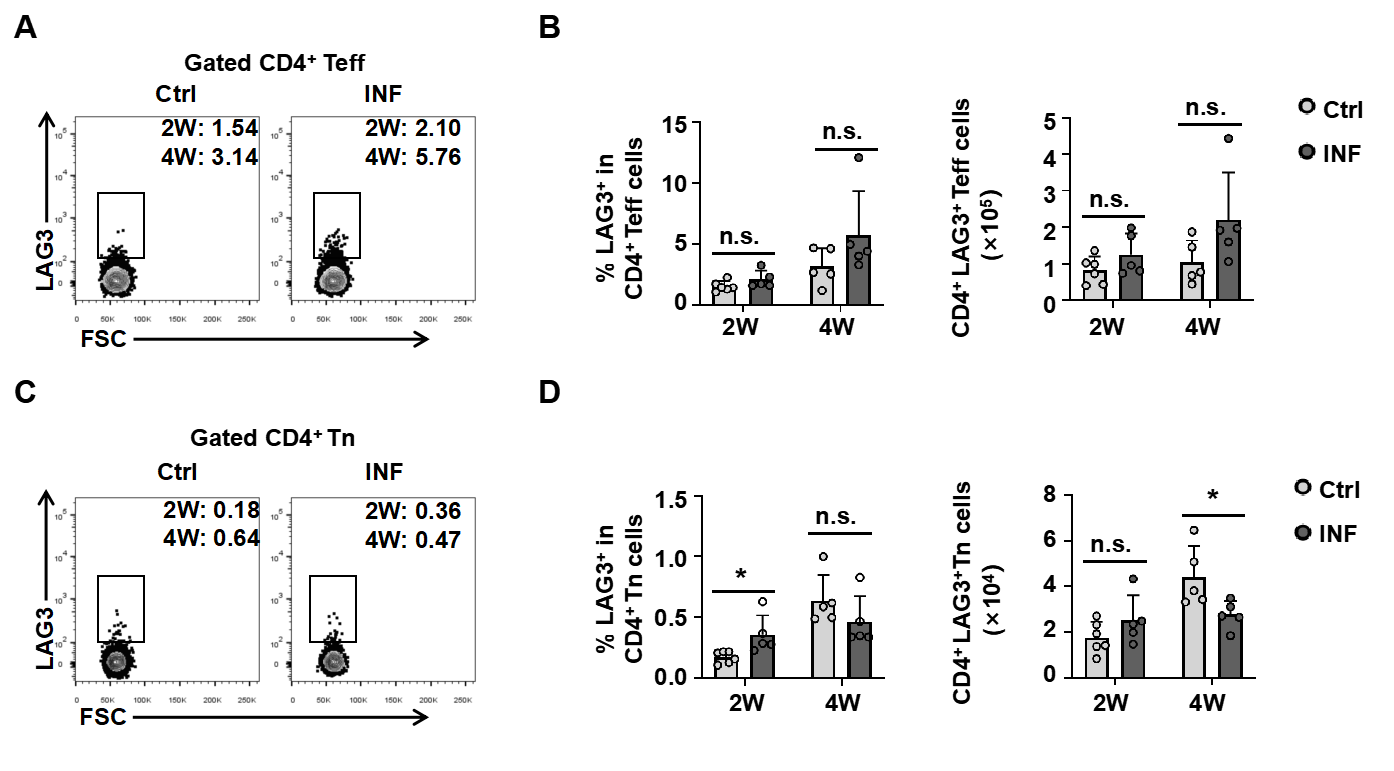
 **Additional file 1: Fig. S 3 LAG3 expression was not up-regulated in CD4^+^ Teff cells in the spleen at the early stages of *E. multilocularis*-infected mice.**(A) Representative flow cytometry plot of LAG3 expression by Teff in CD4^+^ T cells in spleen from Ctrl and INF mice at 2 and 4 weeks. (B) Percentage and absolute numbers of LAG3 expression by Teff in CD4^+^ T cells in spleen from Ctrl and INF mice at 2 and 4 weeks (5-6 mice per group). (C) Representative flow cytometry plot of LAG3 expression by Tn in CD4^+^ T cells in spleen from Ctrl and INF mice at 2 and 4 weeks. (D) Percentage and absolute numbers of LAG3 expression by Tn in CD4^+^ T cells in spleen from Ctrl and INF mice at 2 and 4 weeks (5-6 mice per group). All data are presented as mean + SD. *P < 0.05, n.s., P > 0.05.


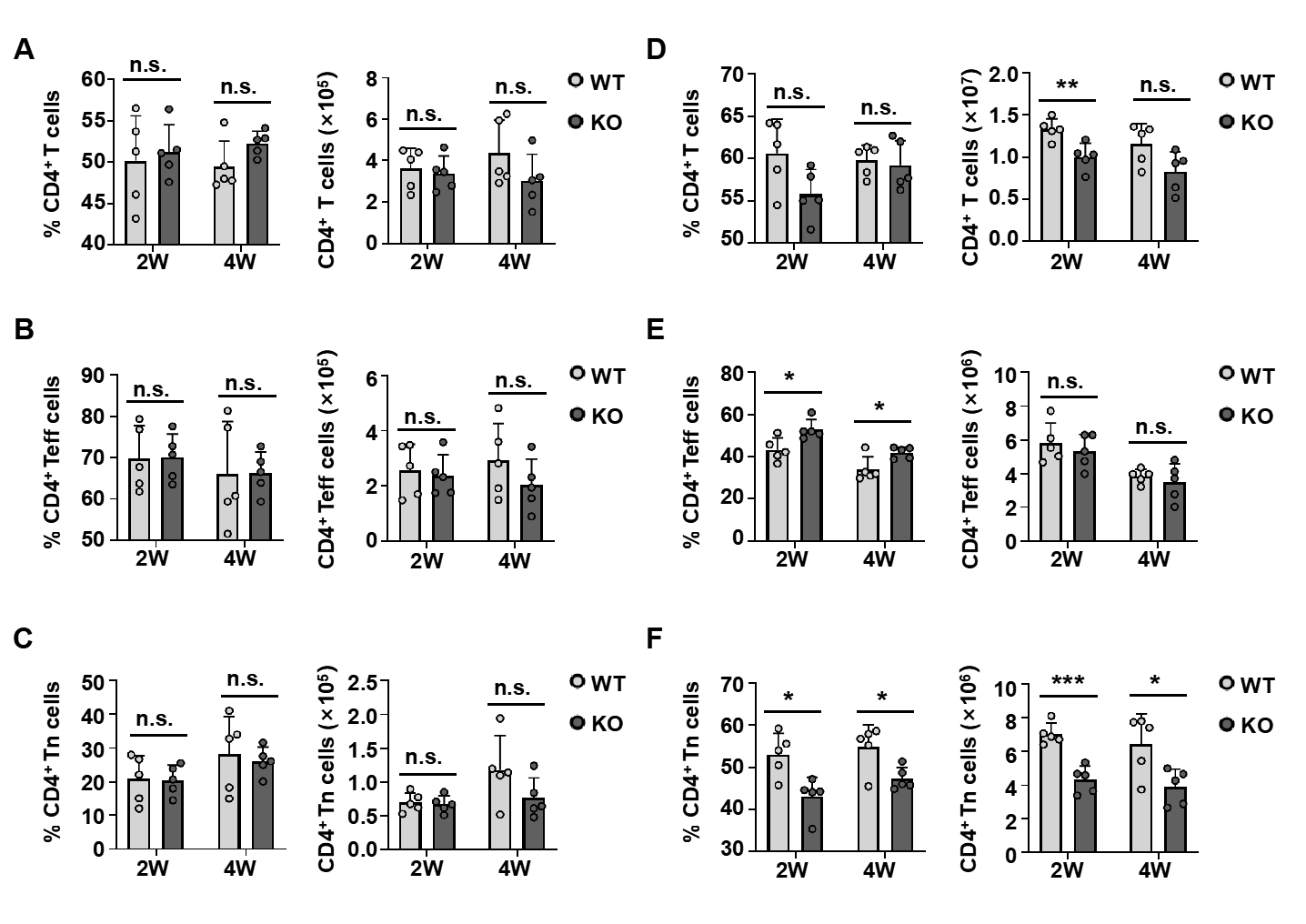
 **Additional file 1: Fig. S 4 LAG3 deficiency promoted CD4^+^ Teff cell differentiation in the spleen at the early stages of *E. multilocularis*-infected mice.**(A) Percentage and absolute numbers of CD4^+^ T cells in the liver from WT and LAG3-KO mice at 2 and 4 weeks of infection (5 mice per group). (B) Percentage and absolute numbers of Teff in CD4^+^ T cells in the liver from WT and LAG3-KO mice at 2 and 4 weeks of infection (5 mice per group). (C) Percentage and absolute numbers of Tn in CD4^+^ T cells in the liver from WT and LAG3-KO mice at 2 and 4 weeks of infection (5 mice per group). (D) Percentage and absolute numbers of CD4^+^ T cells in the spleen from WT and LAG3-KO mice at 2 and 4 weeks of infection (5 mice per group). (E) Percentage and absolute numbers of Teff in CD4^+^ T cells in the spleen from WT and LAG3-KO mice at 2 and 4 weeks of infection (5 mice per group). (F) Percentage and absolute numbers of Tn in CD4^+^ T cells in the spleen from WT and LAG3-KO mice at 2 and 4 weeks of infection (5 mice per group). All data are presented as mean + SD. *P < 0.05, **P < 0.01, ***P < 0.001, n.s., P > 0.05.


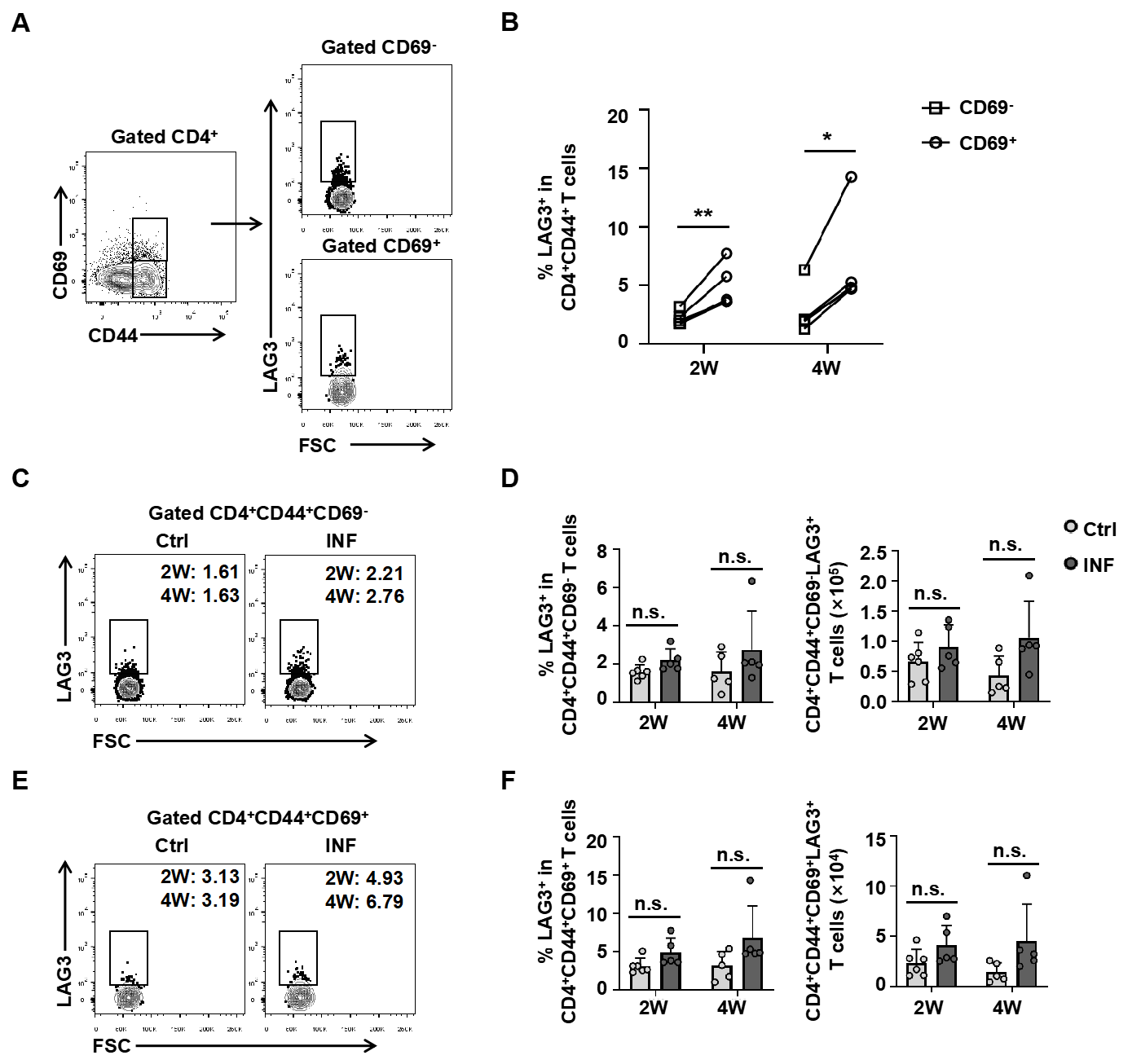


**Additional file 1: Fig. S 5 LAG3 expression was not upregulated on spleen CD4^+^CD44^+^CD69^+^ T cells at the early stages of *E. multilocularis*-infected mice.**(A) Two-dimensional scatterplot, showing the gating strategy used to distinguish between spleen CD4^+^CD44^+^CD69^-^ and CD4^+^CD44^+^CD69^+^ T cells. (B) Percentage of LAG3 expression by CD4^+^CD44^+^CD69^-^ and CD4^+^CD44^+^CD69^+^ T cells in spleen from INF mice after 2 and 4 weeks (5 mice per group). (C) Representative flow cytometry plot of LAG3 expression by CD4^+^CD44^+^CD69^-^ T cells in spleen from Ctrl and INF mice after 2 and 4 weeks. (D) Percentage and absolute numbers of LAG3 expression by CD4^+^CD44^+^CD69^-^ T cells in spleen from Ctrl and INF mice after 2 and 4 weeks (5-6 mice per group). (E) Representative flow cytometry plot of LAG3 expression by CD4^+^CD44^+^CD69^+^ T cells in spleen from Ctrl and INF mice after 2 and 4 weeks. (F) Percentage and absolute numbers of LAG3 expression by CD4^+^CD44^+^CD69^+^ T cells in spleen from Ctrl and INF mice after 2 and 4 weeks (5-6 mice per group). All data are presented as mean + SD. *P < 0.05, **P < 0.01, n.s., P > 0.05.


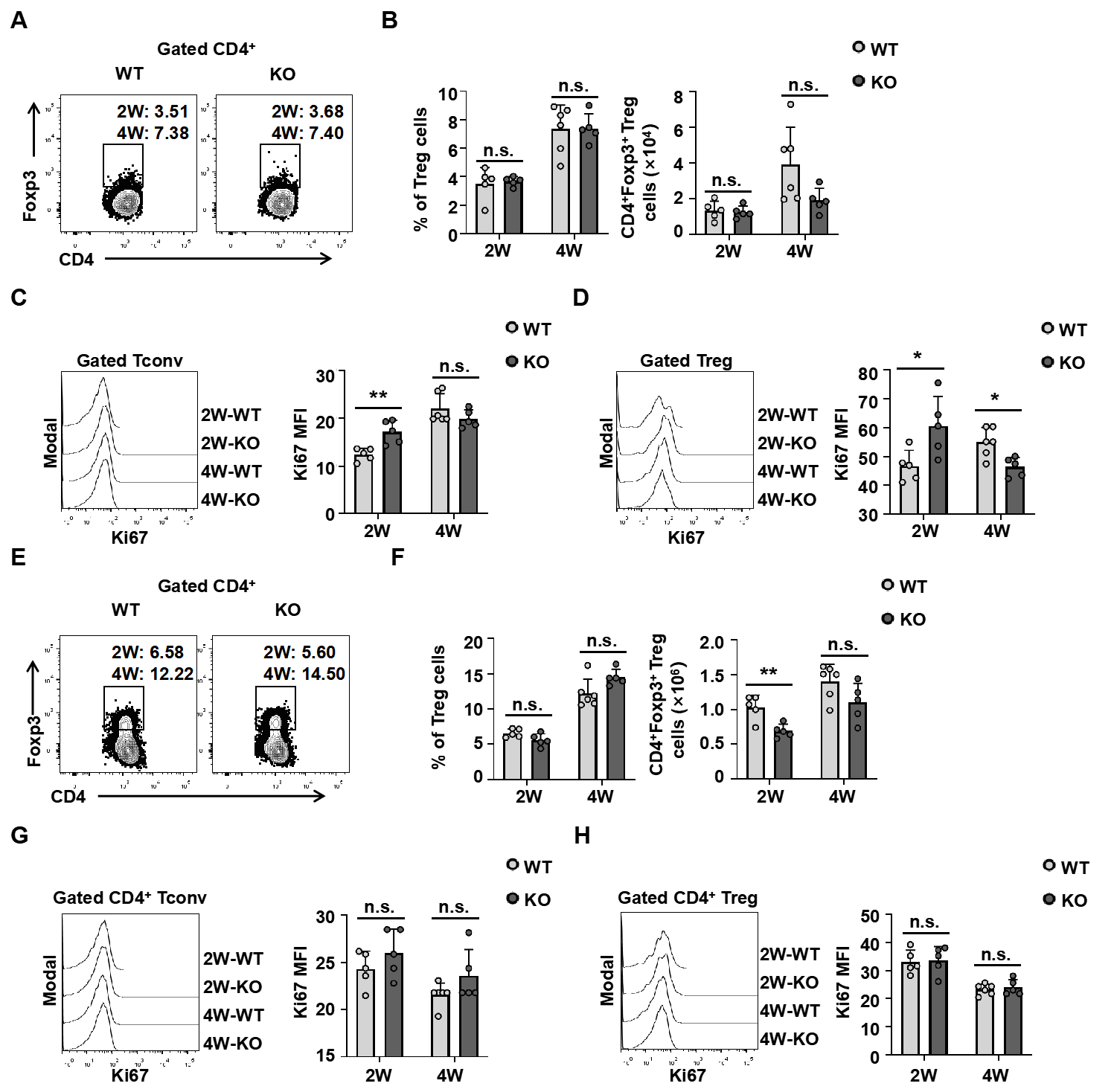


**Additional file 1: Fig. S 6 LAG3 deficiency can regulate the function at early stage in spleen CD4^+^CD44^+^T cells of *E. multilocularis*-infected mice.**(A) Representative flow cytometry plot of Treg cells in the liver from WT and LAG3-KO mice after 2 and 4 weeks of infection (5-6 mice per group). (B) Percentage and absolute numbers of Treg cells in the liver from WT and LAG3-KO mice after 2 and 4 weeks of infection (5-6 mice per group). (C) MFI of Ki67 expression by CD4^+^ Tconv cells in liver from WT and LAG3-KO mice after 2 and 4 weeks of infection (5-6 mice per group). (D) MFI of Ki67 expression by CD4^+^ Treg cells in liver from WT and LAG3-KO mice after 2 and 4 weeks of infection (5-6 mice per group). (E) Representative flow cytometry plot of Treg cells in the spleen from WT and LAG3-KO mice after 2 and 4 weeks of infection (5-6 mice per group). (F) Percentage and absolute numbers of Treg cells in the spleen from WT and LAG3-KO mice after 2 and 4 weeks of infection (5-6 mice per group). (G) MFI of Ki67 expression by CD4^+^ Tconv cells in spleen from WT and LAG3-KO mice after 2 and 4 weeks of infection (5-6 mice per group). (H) MFI of Ki67 expression by CD4^+^ Treg cells in spleen from WT and LAG3-KO mice after 2 and 4 weeks of infection (5-6 mice per group). All data are presented as mean + SD. *P < 0.05, **P < 0.01, ***P < 0.001, n.s., P > 0.05.


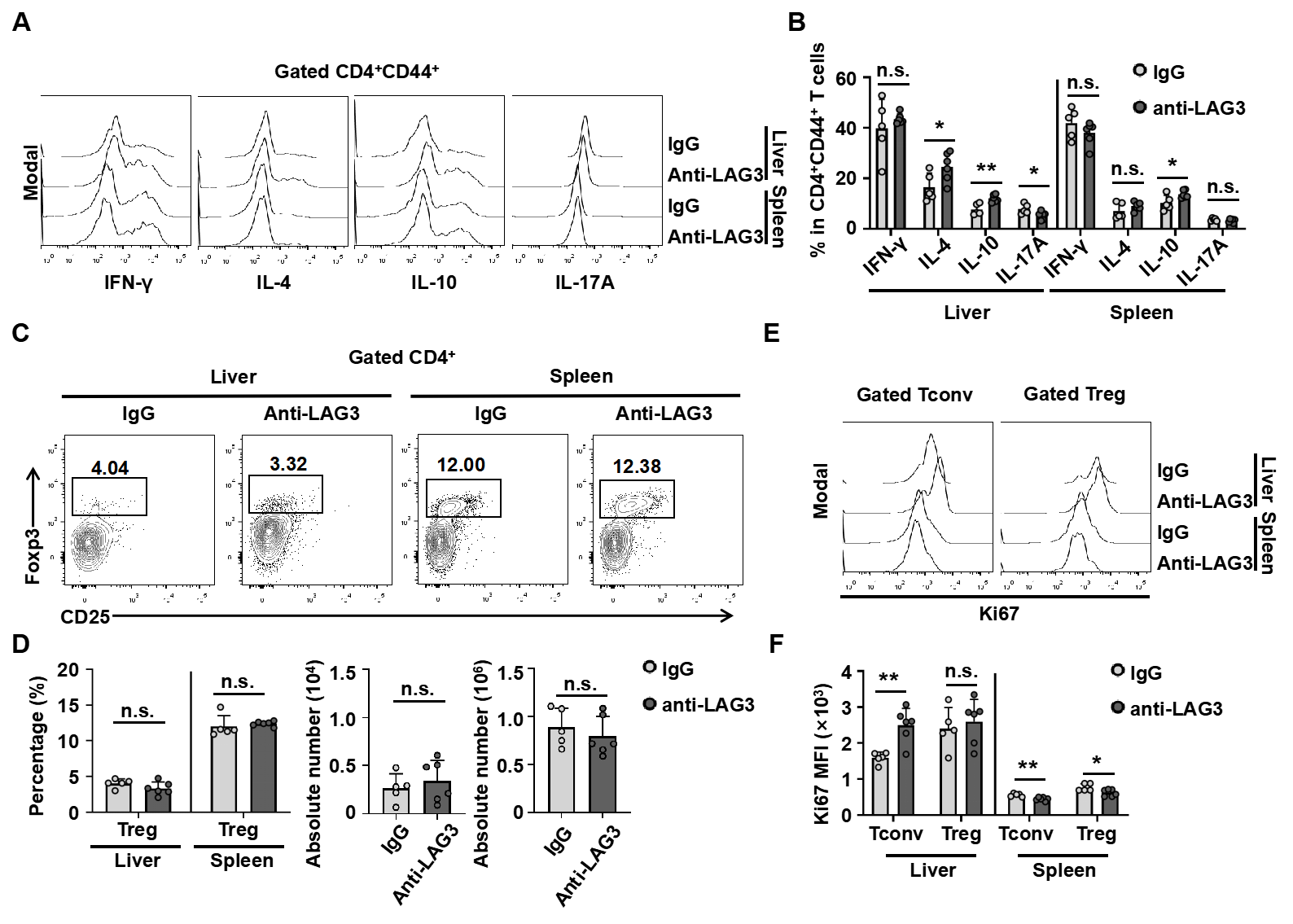


**Additional file 1: Fig. S 7 LAG3 blockade regulates the proliferation of CD4^+^T cells and promotes the production of CD4^+^CD44^+^IL-10^+^ T cells in *E. multilocularis* infected mice.**(A) Representative flow cytometry plot of IFN-γ, IL-4, IL-10, IL-17A production by CD4^+^CD44^+^ T cells in liver and spleen of *E. multilocularis*-infected mice treated with IgG, anti-LAG3 mAb for 4 weeks. (B) Percentage of CD4^+^ T cells, CD4^+^ Tn, CD4^+^ Tem and CD4^+^ Trm in liver and spleen of *E. multilocularis*-infected mice treated with IgG (n=5), anti-LAG3 mAb (n=6) for 4 weeks. (C) Representative flow cytometry plot of Treg cells in the liver and spleen of *E. multilocularis*-infected mice treated with IgG, anti-LAG3 mAb for 4 weeks. (D) Percentage and absolute number of Treg cells in the liver and spleen of *E. multilocularis*-infected mice treated with IgG (n=5), anti-LAG3 mAb (n=6) for 4 weeks. (E) Representative flow cytometry plot of Ki67 expression in Tconv and Treg cells in the liver and spleen of *E. multilocularis*-infected mice treated with IgG, anti-LAG3 mAb for 4 weeks. (F) MFI of Ki67 expression in Tconv and Treg cells in the liver and spleen of *E. multilocularis*-infected mice treated with IgG (n=5), anti-LAG3 mAb (n=6) for 4 weeks. All data are presented as mean + SD. *P < 0.05, **P < 0.01, n.s., P > 0.05.
