# Supplementary material for: LAG3 constrains anti-parasitic response by effector CD4+ T-cell in early Echinococcus multilocularis-infected mice
Source: Parasit Vectors. 2026 Feb 12;19:122. doi: 10.1186/s13071-026-07246-y (PMC12998186; doi:10.1186/s13071-026-07246-y)
Supplement: Supplementary file 2 — Additional file 2. [file 13071_2026_7246_MOESM2_ESM.docx]

**Additional file 1: Table S1. Antibodies for flow cytometry**

| **Reagent or Resource** | **Clone** | **Source** | **Catalog Number** |
| --- | --- | --- | --- |
| Purified anti-mouse CD16/32 |  | BioLegend | Cat# 101302 |
| Anti-mouse CD3-FITC | 17A2 | BioLegend | Cat# 100204 |
| Anti-mouse CD45.1-FITC | A20 | BioLegend | Cat# 110713 |
| Anti-mouse CD11c-FITC | N418 | BioLegend | Cat# 117306 |
| Anti-mouse CD69-PE | H1.2F3 | BioLegend | Cat# 104508 |
| Anti-mouse Ly6c-PE | HK1.4 | BioLegend | Cat# 128008 |
| Anti-mouse IL-4-PE | 11B11 | BioLegend | Cat# 504104 |
| Anti-mouse LAG-3-PE | C9B7W | BioLegend | Cat# 125207 |
| Anti-mouse CD45.2-PE | 104 | BioLegend | Cat# 109808 |
| Anti-mouse MHC Ⅱ-PE/Dazzle 594 | M5/114.15.2 | BioLegend | Cat# 107648 |
| Anti-mouse IL-10-PE/Dazzle 594 | JES5-16E3 | BioLegend | Cat# 505034 |
| Anti-mouse T-bet-PE/Dazzle 594 | 4B10 | BioLegend | Cat# 644828 |
| Anti-mouse CD45.2-PerCP/Cy5.5 | 104 | BioLegend | Cat# 109827 |
| Anti-mouse LAG-3-PerCP/Cy5.5 | C9B7W | BioLegend | Cat# 125212 |
| Anti-mouse Ki67-PerCP/Cy5.5 | 16A8 | BioLegend | Cat# 652424 |
| Anti-mouse CD4-PerCP/Cy5.5 | GK1.5 | BioLegend | Cat# 100433 |
| Anti-mouse CD62L-PerCP/Cy5.5 | MEL-14 | BioLegend | Cat# 104431 |
| Anti-mouse CD45.1-APC | A20 | BioLegend | Cat# 110713 |
| Anti-mouse CD44-APC | IM7 | BioLegend | Cat# 103012 |
| Anti-mouse IFN-γ-APC | XMG1.2 | BioLegend | Cat# 505810 |
| Anti-mouse CD4-APC | GK1.5 | BioLegend | Cat# 100412 |
| Anti-mouse Ly6G- APC/Cyanine7 | 1A8 | BioLegend | Cat# 127624 |
| Anti-mouse CD3- APC/Cyanine7 | 17A2 | BioLegend | Cat# 100222 |
| Anti-mouse CD25- APC/Cyanine7 | PC61 | BioLegend | Cat# 102026 |
| Anti-mouse CD44- APC/Cyanine7 | IM7 | BioLegend | Cat# 103028 |
| Anti-mouse CD69- APC/Cyanine7 | H1.2F3 | BioLegend | Cat# 104526 |
| Anti-mouse CD8- APC/Cyanine7 | 53-6.7 | BioLegend | Cat# 100714 |
| Anti-mouse CD8- Pacific Blue | 53-6.7 | BioLegend | Cat# 100725 |
| Anti-mouse IL17A- Pacific Blue | TC11-18H10.1 | BioLegend | Cat# 506918 |
| Anti-mouse LAG3-Brilliant Violet 421 | C9B7W | BioLegend | Cat# 125221 |
| Anti-mouse Siglec-F-Brilliant Violet 421 | S17007L | BioLegend | Cat# 155509 |
| Anti-mouse CD11b-Brilliant Violet 510 | M1/70 | BioLegend | Cat# 101263 |
| Anti-mouse CD62L-Brilliant Violet 510 | MEL-14 | BioLegend | Cat# 104441 |
| Anti-mouse Foxp3-Alexa Fluor 647 | MF-14 | BioLegend | Cat# 126408 |
| Anti-mouse CD4-Brilliant Violet 605 | GK1.5 | BioLegend | Cat# 100451 |
| Anti-mouse CD19-PE/Cyanine7 | 6D5 | BioLegend | Cat# 115520 |
| Anti-mouse CD45-PE/Cyanine7 | 30-F11 | BioLegend | Cat# 103114 |
| Anti-mouse NK1.1-PE/Cyanine7 | PK136 | BioLegend | Cat# 108714 |
| Anti-mouse LAG3-Brilliant Violet 650 | C9B7W | BioLegend | Cat# 125227 |
| Anti-mouse F4/80-Brilliant Violet 650 | BM8 | BioLegend | Cat# 123149 |
